# Perioperative care based on roy adaptation model in elderly patients with benign prostatic hyperplasia: impact on psychological well-being, pain, and quality of life

**DOI:** 10.1186/s12894-023-01343-1

**Published:** 2023-10-27

**Authors:** Ya-Ni Peng, Lu Jin, E-Jun Peng, Li Zhang

**Affiliations:** Department of Urology, Tongji Hospital, Tongji Medical College, Huazhong University of Science and Technology, Jiefang Avenue 1095, Wuhan, 430030 China

**Keywords:** Benign Prostatic Hyperplasia, Transurethral resection of the prostate, Perioperative Care, Roy Adaptation Model, Health-Related Quality of Life

## Abstract

**Purpose:**

This study aimed to assess the impact of perioperative care based on the Roy Adaptation Model (RAM) on psychological well-being, postoperative pain, and health-related quality of life (HRQoL) in elderly patients with benign prostatic hyperplasia (BPH) undergoing transurethral resection of the prostate (TURP).

**Methods:**

A total of 160 elderly patients diagnosed with BPH between June 2021 and June 2022 and scheduled for TURP were randomly assigned to either the routine care group (n = 80) or the RAM group (n = 80). The RAM group received standard care supplemented with interventions based on the RAM model. Negative emotions measured by the Hospital Anxiety and Depression Scale (HADS), pain intensity by the Visual Analog Scale (VAS), and HRQoL by the 36-Item Short Form Health Survey (SF-36) were measured at the preoperative visit (T0), at 30 days (T1), and at 3 months of (T2) follow‑up.

**Results:**

Repeated measures ANOVA revealed significant differences in psychological well-being, postoperative pain intensity, and HRQoL within both the routine care and RAM groups across the three time points. Holm-Sidak’s multiple comparisons test confirmed significant differences between each time point for both groups. The RAM intervention led to significant reductions in anxiety and depression levels, alleviation of postoperative pain intensity, and improvements in various domains of HRQoL at T1 and T2 compared to routine care.

**Conclusion:**

Incorporating the RAM model into perioperative care for elderly patients undergoing TURP for BPH has shown promising results in improving psychological well-being, reducing postoperative pain intensity, and enhancing HRQoL.

## Introduction

Benign prostatic hyperplasia (BPH) is a prevalent condition among aging men characterized by the enlargement of the prostate gland [1]. It is associated with histological manifestations such as hyperplasia of prostatic stroma and glandular components, leading to anatomically benign prostatic enlargement (BPE) and subsequent bladder outlet obstruction (BOO), resulting in lower urinary tract symptoms (LUTS) [2]. The prevalence of BPH increases with age, affecting approximately 30–40% of individuals in their 40s and reaching around 70–80% in men over the age of 80 [3]. While medical therapy has reduced the need for surgical interventions in the management of BPH, there are cases where patients experience urinary retention and fail subsequent catheter trials, necessitating surgical urological management [4]. Transurethral resection of the prostate (TURP) is the established gold standard procedure primarily used for smaller and medium-sized prostate volumes (up to 80 mL) due to its long-term efficacy based on research evidence [4, 5]. However, TURP is not without risks, as it is associated with intraoperative and postoperative complications such as bleeding, clot retention requiring intervention, genitourinary infections, fluid absorption, and TUR syndrome [6].

Elderly patients undergoing these procedures, particularly those with comorbidities such as cardiovascular disease, hypertension, renal insufficiency, or diabetes mellitus, may experience a potential decline in their quality of life [7]. Therefore, it is crucial to provide perioperative care that addresses not only the physical aspects but also the psychological and social well-being of these patients. In this context, the Roy Adaptation Model (RAM), proposed by nursing scientist Sister Callista Roy in 1970, offers a comprehensive framework to guide nursing care for patients with chronic diseases [8]. The RAM describes behaviors as either adaptive or non-adaptive and focuses on the dynamic process of adaptation in response to four modes: physiological, self-concept, role function, and interdependence [9, 10]. It has been widely applied in various healthcare settings and has demonstrated benefits for promoting adaptation and well-being in elderly patients [11–13]. Therefore, in the context of perioperative care for elderly patients with BPH, the application of the RAM can provide valuable insights and guidance. By incorporating RAM principles into perioperative care, healthcare providers can better understand the unique needs of elderly patients with BPH and implement interventions that address not only their physical health but also their psychological and social well-being.

This study aims to evaluate the impact of perioperative care based on the RAM on negative emotions, postoperative pain, and quality of life in elderly patients with BPH. The findings will contribute to the existing body of knowledge on effective nursing interventions in this population and provide valuable information for improving patient outcomes and enhancing the quality of perioperative care for elderly patients with BPH.

## Methods

### Participants

A total of 160 elderly patients diagnosed with BPH in our hospital between June 2021 and June 2022 were enrolled in this study. They were randomly assigned to two groups using the random number table method: the routine care group (n = 80) and the RAM group (n = 80). Inclusion criteria included age above 65 years, BPH diagnosis, with prostate volume < 80 mL as measured by transabdominal ultrasound, uroflowmetry (measurement of maximum flow rate [Q _max_]) < 12 ml/s or urinary retention, scheduled for TURP, and ability to understand and communicate. Exclusion criteria included neurogenic bladder, bladder neck fibrosis, urethral stricture, thickening of the interureteric ridge, significant comorbidities of cardiovascular, hepatic, or hematopoietic systems, uncontrolled diabetes or diabetic neuropathy, psychiatric disorders, postoperative pathological diagnosis of prostate cancer, severe cognitive impairment, and previous prostate surgery. The study was conducted in accordance with the Declaration of Helsinki (as was revised in 2013), and was approved by Ethics Committee of the Tongji Hospital (TJ-IRB20220940). The informed consent was obtained from each participant prior to enrollment.

### Intervention

Participants in the routine care group received standard preoperative, intraoperative, and postoperative nursing care following the hospital’s established protocols. This care involved conducting necessary preoperative investigations, providing the patient with information about the surgical procedure, monitoring the patient’s vital signs after surgery, maintaining accurate documentation of the monitoring results, ensuring the patient’s incision dressing was dry and clean, maintaining proper skin hygiene, and implementing dietary interventions. Participants in the RAM intervention group received the same standard care as the routine care group but with additional interventions based on the RAM. Throughout the surgical process, a comprehensive approach addresses the patient’s physiological, self-concept, role function, and interdependence need. In the preoperative stage, nurses educate the patient on nutrition and hydration, provide preoperative instructions, and discuss potential physiological impacts. They address fears, encourage expression of concerns, and guide the patient in adapting to changes in daily activities during recovery. In the intraoperative stage, nurses and anesthesiologists provide emotional support and maintain clear communication with the patient. They continuously address emotions, create a supportive environment, and involve the patient in decision-making. Collaboration with the surgical team minimizes disruptions to the patient’ s roles. In the postoperative stage, nurses manage pain and monitor wound healing. They address changes in self-concept, offer psychological support, and assist in adapting to daily activities. Nurses involve family members, fostering interdependence, and suggest community resources for additional support. The RAM intervention incorporated the principles and components of the model into the perioperative care of elderly patients with BPH (Table 1).

**Table 1:** Perioperative care according to the Roy Adaptation Model (RAM)

**Preoperative Stage**	
**Physiological**	1. Educate the patient on the importance of appropriate nutrition and hydration for optimal healing and recovery.
	2. Provide instructions on preoperative preparations, including bowel cleansing and medication management.
	3. Discuss the potential impact of surgery on physiological functions, such as urinary continence and sexual function, and address any concerns.
**Self-concept**	1. Address the patient’s fears, anxieties, and expectations related to the surgical procedure.
	2. Encourage the patient to express their thoughts and concerns about body image and self-perception changes post-surgery.
**Role function**	1. Discuss potential changes in social roles and responsibilities during the recovery period.
	2. Provide guidance on adapting daily activities and routines during the healing process.
**Interdependence**	1. Facilitate the involvement of family members or caregivers in the preoperative education and support process.
	2. Encourage the patient to communicate their needs and concerns to their support network.
**Intraoperative Stage**	
**Physiological**	1. Provide emotional support and reassurance during the preoperative period and upon entering the operating room.
	2. Maintain clear and reassuring communication with the patient during the surgery.
**Self-concept**	1. Continuously address and manage the patient’s concerns, fears, and emotions during the surgical procedure.
	2. Create a calm and supportive environment in the operating room to enhance the patient’s self-concept.
**Role function**	1. Ensure the patient’s active participation and decision-making in the surgical process when possible.
	2. Collaborate with the surgical team to minimize disruptions to the patient’s roles and responsibilities.
**Interdependence**	Involve the patient’s family members or caregivers in the intraoperative process, addressing their concerns and providing updates.
**Postoperative Stage**	
**Physiological**	1. Provide pain management through medication and non-pharmacological approaches.
	2. Monitor wound healing and provide appropriate wound care instructions.
**Self-concept**	1. Address potential changes in body image, self-perception, and self-esteem post-surgery.
	2. Offer psychological support and counseling to address emotional concerns and promote positive self-concept.
**Role function**	1. Assist the patient in adapting to changes in daily activities, including bladder and bowel management.
	2. Provide guidance on resuming social roles and responsibilities post-surgery, including sexual activities if applicable.
**Interdependence**	1. Facilitate support from family members and loved ones, encouraging their involvement in the recovery process.
	2. Suggest community resources, support groups, or peer networks for additional support and social interaction.

### Evaluation of negative emotions, pain, and health-related quality of life (HRQoL)

Data collected at the preoperative visit (T0), at 30 days (T1), and at 3 months of (T2) follow‑up. The HADS (Hospital Anxiety and Depression Scale) was used to evaluate anxiety and depression symptoms, with each subscale having a range of 0–21 [14]. A lower total score indicates lower levels of anxiety and depression, while a higher score suggests more severe symptoms. Pain assessment is typically done using the VAS (Visual Analog Scale) [15], where individuals mark their pain intensity on a scale ranging from “no pain” to the marked point. This distance is converted into a numerical score, often on a scale of 0 to 10, with higher scores indicating greater pain intensity. HRQoL is assessed using the 36-Item Short Form Health Survey (SF-36) [16], which consists of eight dimensions: physical functioning (PF), physical role functioning (PRF), general health perceptions (GHP), vitality (V), social role functioning (SRF), emotional role functioning (ERF), mental health (MH), and Bodily Pain. Scores range from 0 to 100, with higher scores indicating better health or functioning.

### Sample size calculation

In the study design, a meticulous sample size calculation ensured robust statistical power for detecting effects. A existing literature comparing SF36 total scores (Control: 583.05 ± 111.20, RAM: 642.46 ± 75.13) revealed significant improvement with RAM in elderly hypertensive patients [11]. G*Power 3.1.9.2 software determined effect size (d = 0.626). Using a two-tailed test, α = 0.05, and power of 0.95, the calculation suggested 136 participants (68/group). Proactively considering a 15% dropout, 160 participants (80/group) were enrolled, ensuring robust statistical power for reliable results. Additionally, a post-hoc power analysis based on HADS anxiety scores obtained at T2 revealed a mean HADS score of 7.43 ± 4.03 in the routine care group and 5.33 ± 3.34 in the RAM group. The post-hoc analysis, using the “Means: Difference between two independent means” option, yielded a calculated power value of 94.63%. This indicates that the chosen sample size of 80 participants in each group was sufficient to detect the observed effect size of 0.568 with a high level of statistical power.

### Data analyses

The data were analyzed with GraphPad Prism software, setting the significance level at *P <* 0.05. The Shapiro-Wilk test was performed for normal distribution assessment. Parametric tests (independent t-tests) were used for between-group comparisons when data followed a normal distribution (data presented as mean ± standard deviation). In cases where normal distribution was not met, non-parametric Mann-Whitney U tests were utilized, presenting data as median and interquartile range (IQR). Within-group comparisons at different time points used parametric paired t-tests for normally distributed data and non-parametric Wilcoxon matched-pairs signed rank tests for non-normally distributed data. Categorical data were analyzed using Chi-square (*χ*^2^) tests or Fisher’s exact tests.

## Results

### Baseline characteristics of study participants

Baseline characteristics of the study participants are presented in Table 2. The routine care group (n = 80) and the RAM group (n = 80) had comparable baseline characteristics. There were no significant differences between the two groups in terms of age (*P* = 0.787), body mass index (*P* = 0.537), American Society of Anesthesiologists (ASA) score (*P* = 0.139), and various preoperative parameters, including comorbidities, international prostatic symptoms score (IPSS), prostate volume, total operative time, pre-operative Q _max_, as well as the duration of hospital stays and urethral catheter removal (all *P* > 0.05). Regarding postoperative complications, the incidences during the hospital stay and within three months after discharge were comparable between the groups. These complications included fever above 38 °C, clot retention requiring irrigation, clean intermittent catheterization/re-catheterization, pad use on discharge, and readmission during three months (all *P* > 0.05).

**Table 2:** Baseline characteristics of the study participants

	Routine care group (n = 80)	RAM group (n = 80)	*P*
Age (years)	76.0 (71.3 ~ 84.8)	79.0 (71.0 ~ 85.0)	0.787
Body mass index (kg/m^2^)	21.7 (20.6 ~ 23.7)	22.2 (20.3 ~ 23.7)	0.537
ASA	3 (2 ~ 4)	3 (2 ~ 4)	0.139
Preoperative comorbidity			
Diabetes mellitus	10	8	0.803
Oral intake of antiplatelet	11	14	0.664
Preoperative urinary tract infection	9	11	0.812
Preoperative urethral catheter	16	13	0.682
Pre-operative IPSS	19.36 ± 6.38	18.63 ± 7.74	0.512
Prostate volume (ml)	51.74 ± 15.28	50.76 ± 15.55	0.690
Total operative time (min)	75.5 (52.3 ~ 97.0)	75.0 (54.0 ~ 99.8)	0.742
Pre-operative Q _max_ (ml/s)	8.15 (6.23 ~ 9.75)	8.30 (6.80 ~ 9.60)	0.783
Removal of urethral catheter (days)	5 (4 ~ 6)	5 (4 ~ 6)	0.614
Hospital stays (days)	4 (3 ~ 5)	4 (3 ~ 5)	0.621
Postoperative complications			
During stay in hospital			
Fever above 38 °C	5	3	0.720
Clot retention requiring irrigation	3	5	0.720
Clean intermittent catheterization/re-catheterization	5	4	1.000
Pad use on discharge	3	3	1.000
After discharge (within 3 months)			
Fever above 38 °C	6	4	0.746
Pad usage	3	1	0.620
Readmission during 3 months	2	0	0.497

Note: Uroflowmetry (maximum flow rate [Q _max_]); American Society of Anesthesiologists (ASA); International prostatic symptoms score (IPSS); Parametric tests (independent t-tests) were used for between-group comparisons when data followed a normal distribution (data presented as mean ± standard deviation). In cases where normal distribution was not met, non-parametric Mann-Whitney U tests were utilized, presenting data as median and interquartile range (IQR). Categorical data were analyzed using Chi-square (χ2) tests or Fisher’s exact tests

### RAM intervention reduced anxiety and depression in elderly patients with BPH

**Fig. 1:**
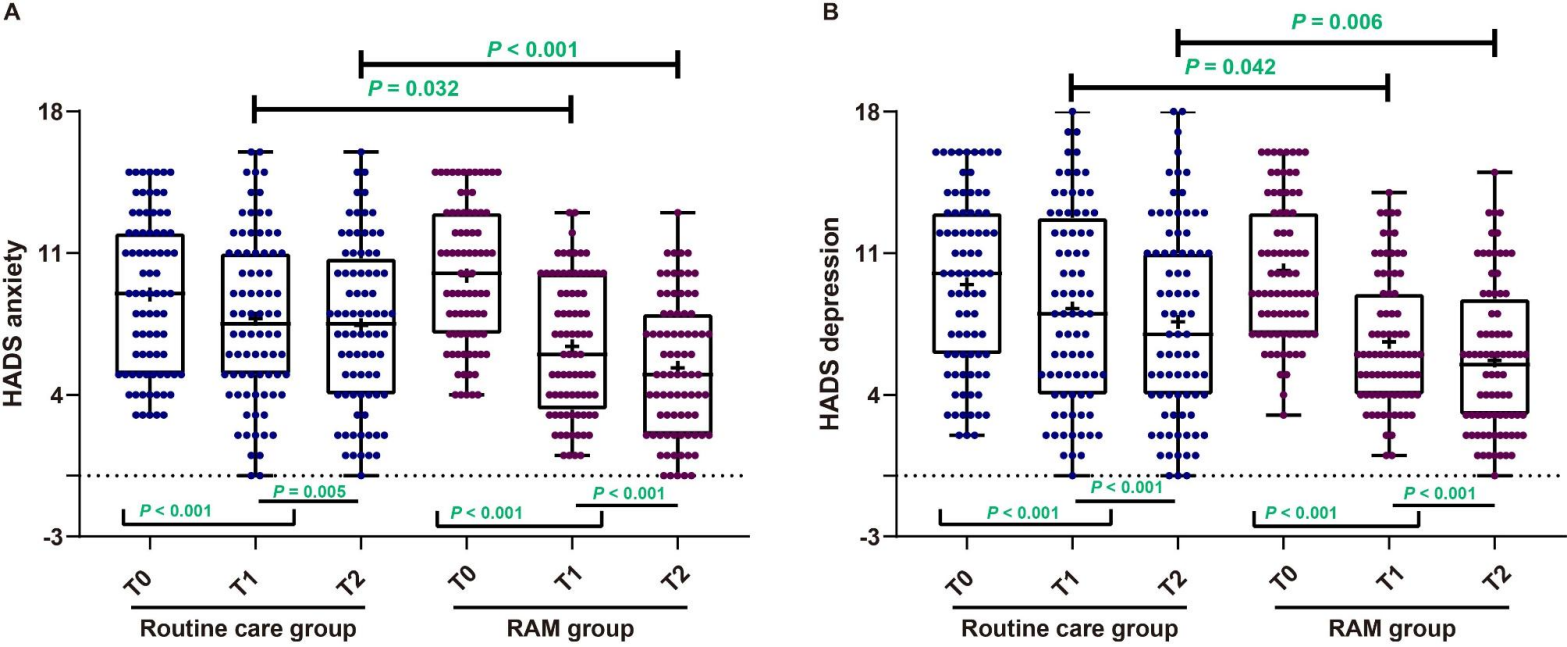
The impact of the RAM intervention on anxiety and depression levels in elderly patients with Benign Prostatic Hyperplasia (BPH), as measured by the HADS scale Note: The results indicate that the RAM intervention significantly reduces anxiety (A) and depression (B) in these patients. The asterisks (*) denote statistical significance, with * indicating *P <* 0.05 and *** indicating *P <* 0.001

The utilization of the Shapiro-Wilk test revealed a non-normal distribution of HADS anxiety and depression scores at three time points for both the routine care group and the RAM group. As illustrated in Fig. 1A and detailed in Table 3, the examination of HADS anxiety score indicated no significant differences between the routine care group and the RAM group at T0 (*P =* 0.110). However, at both T1 and T2, the RAM group exhibited significantly lower scores compared to the routine care group (all *P <* 0.05). In the routine care group, HADS anxiety scores significantly decreased at both T1 (median: 7.5; IQR: 5 ~ 11) and T2 (median: 7.5; IQR: 4 ~ 10.75) compared to T0 (median: 9; IQR: 5 ~ 12, both *P <* 0.001), with a further significant reduction observed at T2 compared to T1 (*P =* 0.005). Similarly, in the RAM group, the HADS anxiety scores at both T1 (median: 6; IQR: 3.25 ~ 10) and T2 (median: 5; IQR: 2 ~ 8) significantly decreased compared to T0 (median: 10; IQR: 7 ~ 13, both *P <* 0.001); furthermore, the T2 score was lower than at T1 (*P <* 0.001). In Fig. 1B; Table 3, assessing the HADS depression score, no significant differences were found between the routine care group and the RAM group at T0 (*P =* 0.338). Nevertheless, at both T1 and T2, the RAM group displayed significantly lower scores compared to the routine care group (all *P <* 0.05). In the routine care group, Wilcoxon tests revealed a substantial reduction in HADS depression scores at both T1 (median: 8; IQR: 4–12) and T2 (median: 7; IQR: 4–11) compared to T0 (median: 10; IQR: 6–13, both *P <* 0.001), with a further significant decrease observed at T2 compared to T1 (*P <* 0.001). Similarly, in the RAM group, HADS depression scores at both T1 (median: 6; IQR: 4–9) and T2 (median: 5.5; IQR: 3-8.75) significantly diminished compared to T0 (median: 9; IQR: 7–13, both *P <* 0.001); moreover, the T2 score was lower than at T1 (*P <* 0.001).

**Table 3:** RAM intervention improved psychological well-being and pain intensity in elderly patients with benign prostatic hyperplasia (BPH) undergoing transurethral resection of the prostate (TURP)

	T0	T1	T2
**HADS anxiety [median (IQR; range)]**			
Routine care group (n = 80)	9 (5 ~ 12; 3 ~ 15)	7.5 (5 ~ 11; 0 ~ 16) ^*^	7.5 (4 ~ 10.75; 0 ~ 16) ^*#^
RAM group (n = 80)	10 (7 ~ 13; 4 ~ 15)	6 (3.25 ~ 10; 1 ~ 13) ^*^	5 (2 ~ 8; 0 ~ 13) ^*#^
*P*	0.110	0.032	< 0.001
**HADS depression [median (IQR; range)]**			
Routine care group (n = 80)	10 (6 ~ 13; 2 ~ 16)	8 (4 ~ 12.75; 0 ~ 18) ^*^	7 (4 ~ 11; 0 ~ 18) ^*#^
RAM group (n = 80)	9 (7 ~ 13; 3 ~ 16)	6 (4 ~ 9; 1 ~ 14) ^*^	5.5 (3 ~ 8.75; 0 ~ 15) ^*#^
*P*	0.338	0.042	0.006
**VAS [median (IQR; range)]**			
Routine care group (n = 80)	6 (5 ~ 8; 3 ~ 10)	6 (4 ~ 7; 1 ~ 9) ^*^	6 (4 ~ 7; 1 ~ 9) ^*#^
RAM group (n = 80)	6.5 (5 ~ 8; 4 ~ 9)	5 (4 ~ 6; 1 ~ 9) ^*^	4 (3 ~ 6; 1 ~ 9) ^*#^
*P*	0.674	0.002	0.002

Note: Hospital Anxiety and Depression Scale (HADS); Visual Analog Scale (VAS); Roy Adaptation Model (RAM); Interquartile Range (IQR). The data was collected at three time points: the preoperative visit (T0), at 30 days (T1), and at 3 months of follow-up (T2). Significant improvements were observed when comparing the data at T0 to both T1 and T2 (* *P* < 0.05). Furthermore, when comparing the data at T1 to T2, there were additional significant improvements (# *P* < 0.05)

### RAM intervention alleviated pain intensity in elderly patients with BPH

**Fig. 2:**
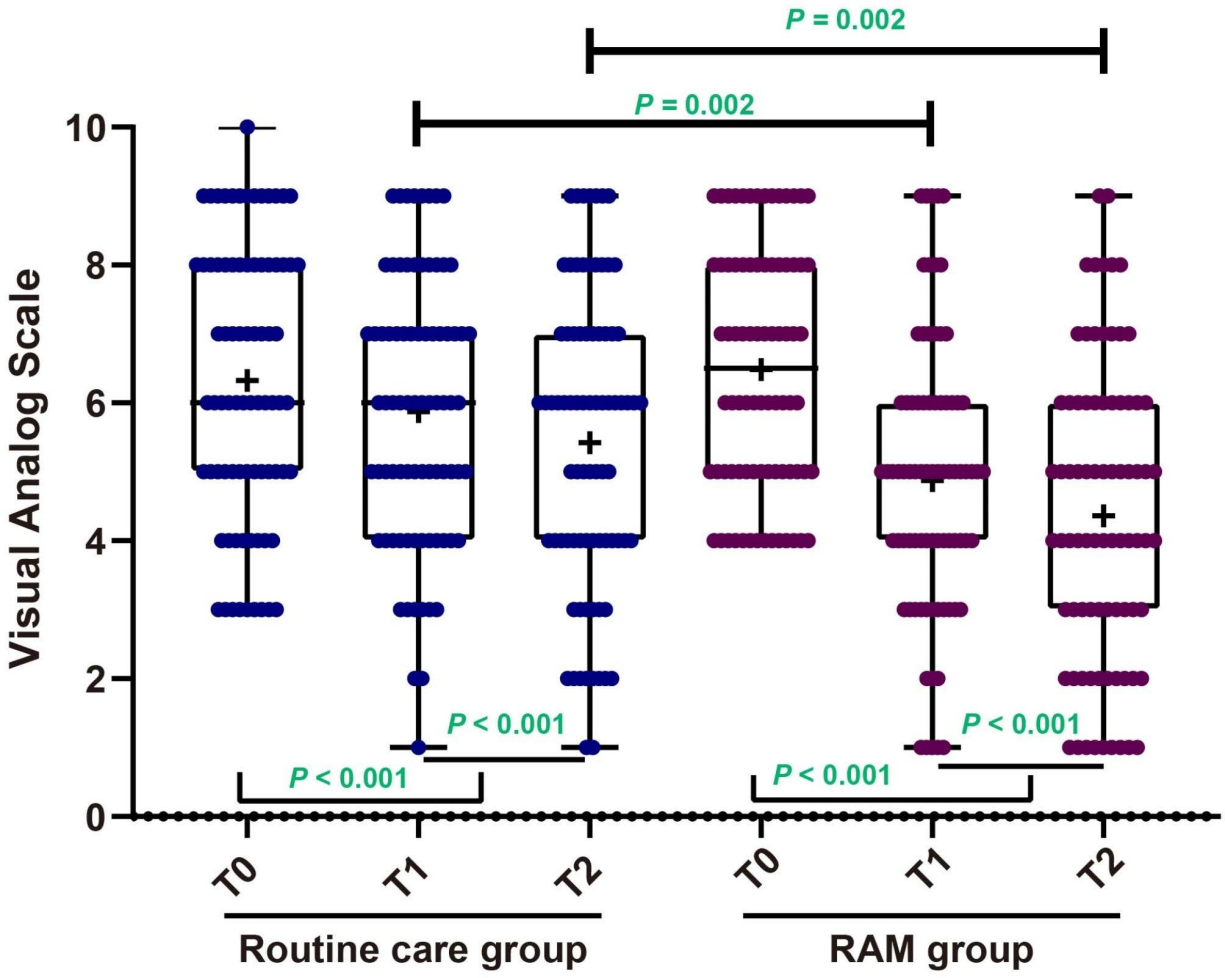
The impact of the RAM intervention on pain intensity in elderly patients with Benign Prostatic Hyperplasia (BPH), as measured by the VAS scale Note: The results indicate that the RAM intervention significantly reduces pain intensity in these patients. The asterisks (*) denote statistical significance, with ** indicating *P <* 0.01 and *** indicating *P <* 0.001

The Shapiro-Wilk test indicated non-normal distribution of VAS scores at all three time points for both the routine care group and the RAM group. As shown in Fig. 2 and outlined in Table 3, in the routine care group, pain intensity scores significantly decreased at T1 (median: 6; IQR: 4–7) and T2 (median: 6; IQR: 4–7) compared to T0 (median: 6; IQR: 5–8, both *P <* 0.001). Moreover, at T2, the VAS score was notably lower than at T1. Likewise, in the RAM group, pain intensity scores at both T1 (median: 5; IQR: 4–6) and T2 (median: 4; IQR: 3–6) significantly decreased compared to T0 (median: 6.5; IQR: 5–8, both *P <* 0.001); furthermore, the T2 score was lower than at T1. These findings underscore a considerable reduction in pain intensity in both groups throughout the study. When comparing the routine care group to the RAM group, Mann-Whitney tests showed no significant difference in pain intensity scores at T0 (*P =* 0.674). However, at both T1 and T2, the RAM group exhibited significantly lower pain intensity scores compared to the routine care group (both *P =* 0.002).

### RAM intervention improved HRQoL in elderly patients with BPH

As demonstrated in Table 4, no significant differences were observed between the routine care group and the RAM group at T0 considering all SF-36 domains (all *P* > 0.05). However, significant improvements were seen in both groups from T0 to T2, indicating that the interventions had a positive impact on these parameters (all *P <* 0.05). Furthermore, when comparing the two groups, the RAM group demonstrated even greater improvements in these SF-36 parameters at T1 and T2, with higher scores in all SF-36 domains (all *P <* 0.05).

**Table 4:** RAM intervention improved health-related quality of life (HRQoL) in elderly patients with benign prostatic hyperplasia (BPH) undergoing transurethral resection of the prostate (TURP)

SF-36 parameters	T0	T1	T2
**Physical functioning (PF)**			
Routine care group	64.84 ± 13.54	69.41 ± 14.06 ^*^	75.29 ± 15.40 ^*#^
RAM group	64.63 ± 14.10	74.06 ± 12.97 ^*^	81.49 ± 11.46 ^*#^
*P*	0.923	0.031	0.004
**Physical role functioning (PRF)**			
Routine care group	60.55 ± 19.71	66.66 ± 17.18 ^*^	75.30 ± 9.15 ^*#^
RAM group	59.40 ± 19.31	72.15 ± 15.61 ^*^	85.39 ± 8.49 ^*#^
*P*	0.710	0.036	< 0.001
**General health perceptions (GHP)**			
Routine care group	63.10 ± 17.02	68.86 ± 16.21 ^*^	75.50 ± 12.29 ^*#^
RAM group	63.43 ± 18.32	73.74 ± 14.30 ^*^	82.30 ± 9.87 ^*#^
*P*	0.908	0.045	< 0.001
**Vitality (V)**			
Routine care group	58.75 ± 15.69	63.24 ± 16.49 ^*^	72.58 ± 13.56 ^*#^
RAM group	59.76 ± 15.94	68.76 ± 15.17 ^*^	79.29 ± 10.90 ^*#^
*P*	0.686	0.029	< 0.001
**Social role functioning (SRF)**			
Routine care group	67.55 ± 16.86	68.10 ± 19.08	79.88 ± 9.97 ^*#^
RAM group	66.10 ± 16.42	74.76 ± 13.64 ^*^	83.80 ± 8.11 ^*#^
*P*	0.582	0.012	0.007
**Emotional role functioning (ERF)**			
Routine care group	60.54 ± 19.46	66.19 ± 16.44 ^*^	72.60 ± 11.39 ^*#^
RAM group	57.98 ± 18.29	71.31 ± 14.49 ^*^	83.65 ± 10.15 ^*#^
*P*	0.392	0.038	< 0.001
**Mental health (MH)**			
Routine care group	70.18 ± 13.51	72.96 ± 15.02 ^*^	80.21 ± 9.40 ^*#^
RAM group	70.59 ± 13.17	77.8 ± 10.75 ^*^	85.63 ± 6.85 ^*#^
*P*	0.845	0.020	< 0.001
**Bodily pain**			
Routine care group	64.91 ± 14.55	70.65 ± 14.79 ^*^	81.68 ± 7.23 ^*#^
RAM group	64.53 ± 15.35	76.05 ± 12.97 ^*^	87.19 ± 7.78 ^*#^
*P*	0.870	0.015	< 0.001

Note: The data presented as mean ± standard deviation was collected at three time points: the preoperative visit (T0), at 30 days (T1), and at 3 months of follow-up (T2). Significant improvements were observed when comparing the data at T0 to both T1 and T2 (* *P* < 0.05). Furthermore, when comparing the data at T1 to T2, there were additional significant improvements (# *P* < 0.05)

## Discussion

Elderly patients undergoing surgical interventions for BPH often experience various stressors, including anxiety, postoperative pain, and disruptions to their quality of life [4]. The RAM is a nursing model commonly used in nursing research to assess clients’ adaptation to a changing environment [9, 17]. In this study, we examined the impact of RAM-based perioperative care in elderly patients with BPH, a population with specific care needs and considerations. The positive outcomes observed in terms of anxiety and depression reduction, pain management, and improvement in HRQoL suggest that implementing a holistic and patient-centered approach, such as the RAM framework, may be beneficial in this population.

Our findings regarding anxiety and depression align with previous research on RAM-based interventions in psychological well-being. For instance, a study by Yu Z et al. investigated the impact of RAM-based perioperative care on anxiety and depression in patients with early-stage lung carcinoma undergoing radical resection and reported significant reductions in anxiety and depression levels postoperatively. This suggests that the RAM framework effectively addresses the psychological distress associated with surgical procedures [18]. Similarly, studies in patients undergoing bariatric surgery and instrumental reminiscence therapy based on RAM have demonstrated positive effects on physical activity, energy, adaptation, life satisfaction, and happiness [19, 20]. Consistent with these findings, our study showed a significant decrease in anxiety and depression levels among elderly patients with BPH who received the RAM intervention.

Regarding postoperative pain management, our study supports previous research highlighting the benefits of RAM-based interventions. RAM considers various adaptive modes that influence people’s responses to pain, making it applicable in pain management [13]. Additionally, a scoping review on chronic pain and adaptation processes in older adults with persistent pain concluded that RAM is appropriate for explaining these processes and is more applicable in clinical practice [17]. Our study demonstrated a significant decrease in pain intensity in elderly BPH patients who received the RAM intervention, further emphasizing the relevance of RAM-based perioperative care in optimizing pain control.

In terms of HRQoL, our findings are consistent with studies that explored the impact of RAM-based interventions on various domains of HRQoL. For example, nursing interventions based on RAM have been shown to enhance self-efficacy, self-management, healthy behavior changes, medication compliance, and blood pressure control in elderly hypertensive patients, resulting in improved HRQoL [11]. Esmaili, M et al. conducted a study on the effects of RAM-based perioperative care on HRQoL in patients undergoing coronary artery bypass graft surgery and reported decreases in fatigue levels and improvements in HRQoL [21]. Similarly, our study demonstrated significant improvements in SF-36 parameters among elderly BPH patients who received the RAM intervention. These findings underscore the positive impact of RAM-based perioperative care on overall well-being and HRQoL outcomes.

While prior research has explored RAM-based interventions across diverse patient populations and surgical contexts, its application specifically in elderly BPH patients requires further investigation. Subsequent studies should compare outcomes of RAM-based perioperative care with established models in similar surgical interventions for elderly BPH patients, enhancing understanding of RAM’s unique benefits in this demographic. Additionally, assessing the long-term effects of RAM-based perioperative care, beyond our study’s scope, is crucial. Examining patient outcomes and quality of life measures, like six months or one-year post-surgery, can reveal sustained benefits and impacts on long-term recovery and well-being in elderly BPH patients. Despite the promising results favoring RAM-based care, adopting a new patient management strategy necessitates careful consideration. Factors such as potential additional time demands on healthcare professionals, training requirements, and adjustments in resource allocation should be meticulously weighed. A comprehensive cost-benefit analysis is essential to evaluate the economic implications of transitioning to RAM-based care. While our study underscores positive outcomes, practical feasibility and sustainability in real-world clinical settings demand thorough evaluation.

In conclusion, our study contributes to the growing evidence supporting the effectiveness of perioperative care based on the RAM in elderly patients with BPH. The findings align with previous research demonstrating reductions in anxiety and depression, improved pain management, and enhanced HRQoL outcomes with RAM-based interventions. By implementing a holistic and patient-centered approach, healthcare professionals can optimize the well-being and quality of life of elderly BPH patients undergoing surgical treatment.

## Data Availability

The datasets used and/or analyzed during the current study are available from the corresponding author on reasonable request.

## References

[1] Arnold MJ, Gaillardetz A, Ohiokpehai J (2023). Benign Prostatic Hyperplasia: Rapid evidence review. Am Fam Physician.

[2] Zeng XT, Jin YH, Liu TZ, Chen FM, Ding DG, Fu M, Gu XQ, Han BM, Huang X, Hou Z (2022). Clinical practice guideline for transurethral plasmakinetic resection of prostate for Benign Prostatic Hyperplasia (2021 Edition). Mil Med Res.

[3] Madersbacher S, Sampson N, Culig Z (2019). Pathophysiology of Benign Prostatic Hyperplasia and Benign Prostatic Enlargement: a Mini-review. Gerontology.

[4] Hartley B (2014). Older patient perioperative care as experienced via transurethral resection of the prostate (TURP). J Perioper Pract.

[5] Miernik A, Gratzke C (2020). Current treatment for Benign Prostatic Hyperplasia. Dtsch Arztebl Int.

[6] Yilmaz M, Esser J, Suarez-Ibarrola R, Gratzke C, Miernik A (2022). Safety and Efficacy of Laser Enucleation of the prostate in Elderly patients - A Narrative Review. Clin Interv Aging.

[7] Piao S, Choo MS, Kim M, Jeon HJ, Oh SJ (2016). Holmium Laser Enucleation of the prostate is safe for patients above 80 years: a prospective study. Int Neurourol J.

[8] Alimohammadi N, Maleki B, Abbasi S, Shakerian B, Hemati Z (2018). The Effect of Adaptation Training on Controlling Maladaptation behaviors in adolescents with Asthma based on Roy Adaptation Model. Tanaffos.

[9] Goudarzi F, Khadivzadeh T, Ebadi A, Babazadeh R (2022). Women’s interdependence after hysterectomy: a qualitative study based on Roy adaptation model. BMC Womens Health.

[10] Klotzbaugh R, Fawcett J (2023). Gender minority persons’ perceptions of peer-led support groups: a Roy Adaptation Model Interpretation. ANS Adv Nurs Sci.

[11] Zhang J, Guo L, Mao J, Qi X, Chen L, Huang H, Sun Y, Yang X (2021). The effects of nursing of Roy adaptation model on the elderly hypertensive: a randomised control study. Ann Palliat Med.

[12] Salazar-Barajas ME, LilloCrespo M, Hernandez Cortez PL, Villarreal Reyna MLA, Gallegos Cabriales EC, Gomez Meza MV. Salazar Gonzalez BC: Factors Contributing to Active Aging in Older Adults, from the Framework of Roy’s Adaptation Model. Invest Educ Enferm 2018, 36(2). 10.17533/udea.iee.v36n2e08 30148942

[13] Flanagan NM (2018). Persistent Pain in older adults: Roy’s adaptation model. Nurs Sci Q.

[14] de Oliveira L, Souza EC, Rodrigues RAS, Fett CA, Piva AB (2019). The effects of physical activity on anxiety, depression, and quality of life in elderly people living in the community. Trends Psychiatry Psychother.

[15] Suzuki Y, Iijima H, Tashiro Y, Kajiwara Y, Zeidan H, Shimoura K, Nishida Y, Bito T, Nakai K, Tatsumi M (2019). Home exercise therapy to improve muscle strength and joint flexibility effectively treats pre-radiographic knee OA in community-dwelling elderly: a randomized controlled trial. Clin Rheumatol.

[16] Lera L, Marquez C, Saguez R, Moya MO, Angel B, Albala C (2021). [Quality of life of older people with depression and dependence: validity of the SF-12 (short form health survey) questionnaire]. Rev Med Chil.

[17] Nawai A (2019). Chronic Pain Management among older adults: a scoping review. SAGE Open Nurs.

[18] Yu Z, Jia W, Sun X, Zhang S, Tan J, Feng L (2023). Effect of Roy’s adaptation, Model-based, Perioperative Nursing Service on patients: a clinical observational study. Altern Ther Health Med.

[19] Kabu Hergul F, Ozbayir T, I Am As Normal As Everyone Now (2021). Examination of experiences of patients undergoing bariatric Surgery according to Roy’s adaptation model: a qualitative study. Clin Nurs Res.

[20] Aydogdu O, Tastan S, Kublay G (2023). The effects of the instrumental reminiscence therapy based on Roy’s adaptation model on adaptation, life satisfaction and happiness in older people: a randomized controlled trial. Int J Nurs Pract.

[21] Esmaili M, Salehi-Tali S, Mazaheri E, Hasanpour-Dehkordi A, Kheiri S (2022). The Effect of the nursing care based on the Roy Adaptation Model on the level of the quality of life and fatigue in the patients undergoing coronary artery bypass graft Surgery. Crit Care Nurs Q.

